# Environmental and spatial determinants of enteric pathogen infection in rural Lao People’s Democratic Republic: A cross-sectional study

**DOI:** 10.1371/journal.pntd.0008180

**Published:** 2020-04-08

**Authors:** Anna N. Chard, Karen Levy, Kelly K. Baker, Kevin Tsai, Howard H. Chang, Vonethalom Thongpaseuth, Jeticia R. Sistrunk, Matthew C. Freeman

**Affiliations:** 1 Gangarosa Department of Environmental Health, Rollins School of Public Health, Emory University, Atlanta, Georgia, United States of America; 2 Department of Occupational and Environmental Health, College of Public Health, University of Iowa, Iowa City, Iowa, United States of America; 3 Department of Biostatistics and Bioinformatics, Rollins School of Public Health, Emory University, Atlanta, Georgia, United States of America; 4 Laboratory and Treatment Unit, Center for Malariology, Parasitology, and Entomology, Ministry of Health, Vientiane, Lao PDR; Chinese Center for Disease Control and Prevention, CHINA

## Abstract

Though the health risks associated with poor access to water, sanitation, and hygiene (WASH) are well established, recent large-scale WASH trials have found limited impact on enteric disease. The aims of this study were to: 1. estimate the prevalence of enteropathogens among children <5, school-aged children, and adults; 2. model associations between WASH transmission pathways and enteropathogen infections; and 3. quantify clustering of enteropathogen infections at the household- and village-level. We conducted a cross-sectional survey in 50 villages in Saravane Province, Lao People’s Democratic Republic. From 297 households, we collected 891 fecal samples from one child <5, one school-aged child, and one adult living in the same household, and collected survey and observational data on household demographics, WASH access, and animal ownership. Fecal samples were analyzed for 25 enteropathogens using a qRT-PCR assay. We observed near universal infection with at least one enteropathogen (98.3%). Few household or village-level WASH covariates were statistically associated with enteropathogen infection. Concordant household infection was higher than expected under the independence assumption for 14 of the 21 pathogens for which we had sufficient data, indicating strong household correlation for many infections. Median odds ratios (MORs), a measure of cluster-level (e.g. village and household) influence on an individual’s odds of infection, were elevated at the village level, particularly for viruses (MOR: 3.89; 95% confidence interval [CI]: 2.64, 6.69), protozoa (MOR: 2.07; 95% CI: 1.59, 3.10), and soil-transmitted helminths (MOR: 2.46; 95% CI: 1.89, 3.56), indicating strong village-level differences in individuals' odds of enteric infections. WASH access, as hypothesized, is associated with fewer enteroinfections, but WASH access as currently defined does not reveal a measurably protective association with infection for many etiologies. Household- and community-level factors beyond WASH access, such as intra-household pathogen transmission, exposure to animal feces, and contextual factors in the public domain may be important risk factors for enteric infections.

**Trial registration:**

clinicaltrials.gov (NCT02342860).

## Author summary

Despite clear linkages between poor access to water, sanitation, and hygiene (WASH) and risk of enteric diseases, there is mixed evidence of the ability of WASH interventions to mitigate these diseases based on findings from the “gold standard” randomized controlled trials. This study examines drivers of enteric diseases by exploring associations between household- and community-level WASH access and enteropathogen infections. We found that enteropathogen infections were nearly universal across age groups, with 98.3% of subjects having at least one infection. Few household or village-level WASH covariates were statistically associated with enteropathogen infection; WASH access generally trended towards lower infection, though trends varied by transmission pathway. Factors other than WASH access, such as intra-household transmission, animal feces exposure, and environmental contamination may be important risk factors for enteric infections. These results highlight the challenges of addressing enteric infections using many of the existing WASH intervention approaches.

## Introduction

The health risks associated with inadequate access to water, sanitation, and hygiene (WASH) are well documented [1–3]. Interventions to improve water, sanitation, and hygiene are associated with 33%, 25%, and 30% reductions in risk of childhood diarrhea, respectively [4], and improved WASH is associated with reductions in *Giardia* [5] and neglected tropical diseases (NTDs) such as soil-transmitted helminths (STHs), trachoma, and schistosomiasis [6–9]. Yet, several recent, high-profile impact evaluations have failed to demonstrate a significant reduction from WASH on stunting and diarrhea [10–14], STH infection [10, 13], and other enteric infections [15].

Enteric infections and diarrheal diseases may be caused by over 40 pathogens, shed in both human and animal feces, many of which can persist in environmental reservoirs, with different etiologies and dominant transmission pathways [16–20]. A critical transmission route across all enteropathogen taxa (defined here as virus, bacteria, protozoa, or STH) is the fecal-oral route, i.e., consumption of fecally contaminated food and water and interaction with fecally contaminated environments (i.e., soil and surface water) [21, 22], as traditionally depicted by the F-diagram [23].

One hypothesis for the inconclusive findings of recent WASH trials is that interventions are not sufficiently targeting the relevant transmission pathways for the most prevalent pathogens. For example, to improve drinking water quality, the WASH Benefits trial promoted water chlorination [12], which is ineffective against *Cryptosporidium* spp. [24], one of the leading causes of moderate-to-severe diarrhea among young children [25]. Another hypothesis is that many WASH trials are designed to prevent exposure to human feces and do not adequately address exposure to animal feces [26, 27]. Animal feces present a substantial risk to human health, as animals may be the leading driver of pathogen diversity in the environment [21] and many enteropathogens that cause moderate to severe diarrhea are of animal origin [26].

A further consideration of challenges to the effectiveness of WASH interventions is that they are often implemented at the household, rather than the community-level [10–12, 28]. Indeed, households are important loci of WASH-related disease transmission, since domestic activities and behaviors can result in the sharing of infective sites, thus leading to similar risks of infection among household members [29, 30]. However, sanitation may provide community-level, or herd, protection on health outcomes such as diarrhea, trachoma, nutritional status, and infant mortality but only if high levels of coverage are achieved [31–39]. Open defecation or inadequately managed sanitation resulting in environmental contamination of fecal sludge can increase direct and indirect contact with fecal contamination through soil, surface water, and feces in public settings [21, 40], which can lead to ingestion of enteric pathogens [41], even among households with toilets [36, 42].

Here we describe the prevalence and distribution of WASH-related enteric pathogens in the context of household and community WASH access. We conducted a cross-sectional analysis of fecal samples collected from children <5, school-aged children, and adults residing in the same households in rural Lao People’s Democratic Republic (Lao PDR, Laos). The aims of this study were to 1. estimate the prevalence of enteropathogens among children <5, school-aged children, and adults and identify differences by age group; 2. model associations between WASH transmission pathways, including household- and community-level WASH access and exposure to animal feces, and enteropathogen infections at the taxa- and pathogen-level (taxa defined here as bacteria, virus, protozoa, or STH); and 3. quantify clustering of enteropathogen infections at the household- and village-level.

## Methods

### Setting

This cross-sectional study was nested within the Water, Sanitation, and Hygiene for Health and Education in Laotian Primary Schools (WASH HELPS) study, a longitudinal cluster-randomized trial evaluating a comprehensive school-based water, sanitation, and hygiene (WASH) intervention in 100 schools in Saravane Province, Lao PDR between September 2014 and May 2017. Detailed methods of the parent study are described elsewhere [43, 44]. The WASH HELPS study is registered at clinicaltrials.gov (NCT02342860).

### Ethics

This study was approved by Emory University’s Institutional Review Board (IRB0076404) and the National Ethics Committee for Health Research (Ref.no. 043/NECHR), Ministry of Health, Lao PDR. Adult participants provided informed verbal consent for the household survey and stool collection for themselves and their children prior to any data collection. Verbal consent was used in lieu of written consent due to varying levels of literacy as well as a desire to minimize paperwork containing participants’ names. Study staff documented consent on a separate master list and used a unique household and participant code for survey and stool data. Both ethics committees approved the use of verbal consent.

### Study design

Methods of this sub-study are described in detail elsewhere [45]. Briefly, we selected 50 of the 100 school-hosting villages participating in the WASH HELPS study using stratified random sampling based on district and WASH HELPS study intervention status. In each village, we randomly selected 25 households meeting two eligibility criteria: 1) having a child attending the primary school participating in the WASH HELPS study, and 2) having a child <5 years old living in the household. We conducted a household survey to collect information on household demographics, asset and animal ownership, recent illness among household members, and WASH access and behaviors. We also conducted structured observations of WASH facilities when present.

During the household survey, we distributed three pre-labeled, resealable plastic bags, each containing a plastic spoon to collect stool samples from the pupil, the pupil’s parent/caregiver (preference was given to female parent/caregiver due to evidence of mothers’ hand contamination as an important vector for household disease transmission [46]), and the pupil’s sibling <5 years old (if multiple siblings, preference was given to youngest sibling). Participants were instructed to collect the first stool on the following morning. Stool samples were collected in the morning and transported with a cold chain to the field laboratory within two hours of collection. A second return visit was made the following day if households did not return all three participants’ stool samples on the first day. All data were collected between February-April 2017 (dry season), prior to annual school-based chemotherapy for STH. The time frame corresponded with the final round of data collection and conclusion of the WASH HELPS study [43, 44].

For this sub-study, a second subset of households (n = 297) were selected from those in the first sub-study for additional enteropathogen analysis using stratified random sampling based on district and WASH HELPS intervention status. Sample size was based on the maximum number of households we could include given the study budget. Households were eligible for inclusion only if all three subjects in the household (adult, school-aged child, and child <5 years old, “household triad”) returned their stool sample on the same day. Including multiple subjects from the same household allowed us to quantify household-level clustering of infection and distinguish village-level effects from household-level effects.

### Laboratory analysis

Laboratory procedures have been described in detail elsewhere [45]. Briefly, in the field laboratory we aliquoted 200 mg of stool into a DNA/RNA Shield Collection and Lysis Tube (Zymo Research, Irvine, CA, USA). One field control was processed each day using DNA/RNA-free water to evaluate the possibility of false positives from contamination in the field laboratory during sampling. Samples were kept frozen at -20°C (between 6 and 41 days) until transported to a laboratory at Emory University, where they were subsequently stored at -80°C until further processing. Total nucleic acid was extracted from samples using the ZymoBIOMICS DNA/RNA Mini Kit (Zymo Research, Irvine, CA, USA). One extraction blank was included per batch to exclude the possibility of false positives from contamination during extraction. Extractions were transported on dry ice to the University of Iowa and analyzed on a ViiA7 thermocycler (Thermo Fisher, Carlsbad, CA, USA) via a 40 cycle quantitative reverse transcription polymerase chain reaction (qRT-PCR) analysis using a custom TaqMan Array Card (TAC) (Thermo Fisher, Carlsblad, CA, USA) with compartmentalized, probe-based qPCR assays for 25 enteropathogens [47, 48]. TAC primer and probe sequences are listed in **S1 Table**. TAC preparation was based on the protocol described by Liu et al., with the exception of including 0.3 μM BSA to reduce inhibition of nucleic acid amplification [47].

Two researchers manually read TAC data; a third researcher resolved conflicting results. Multicomponent plots were inspected for increases in fluorescence for the FAM-based gene-specific probe to validate true amplification of the complete gene target. No false positive signals were observed for any pathogen gene in 25 water-only qPCR controls within 40 cycles of amplification. The limit of detection for all assays using the TAC card have been experimentally determined to lie between 33 and 40 cycle thresholds, as described elsewhere. Thus, a sample was initially classified as positive for a pathogen if amplification was detected for one or both duplicates of a gene assay. Samples were ultimately considered positive only if the corresponding field and extraction blanks were negative, otherwise the data were considered invalid [48].

### Measures

In the primary analysis, the infection outcome variables were defined by presence/absence of any viral, bacterial, protozoal, or STH gene marker. In the secondary analysis, the outcome variables were presence/absence of each individual pathogen.

The exposure variables included household-level improved drinking water source (reported), improved sanitation facility (observed), and basic handwashing facility (observed), all classified according to WHO/UNICEF Joint Monitoring Programme standards [49]; animal ownership, which was reported as owning any cows, goats, sheep, poultry (chickens or ducks), or pigs; and village-level prevalence of an improved drinking water source (“improved drinking water coverage”), an improved sanitation facility (“improved sanitation coverage”), and a basic handwashing facility ("basic handwashing facility coverage"). Village-level WASH prevalence was calculated by aggregating household-level WASH access variables of all households in the sub-study at the village-level (cluster), excluding each individual’s own household in order to better represent indirect exposure and to avoid forced correlation between household- and village-level covariates [31]. Village-level WASH prevalence was re-scaled with cut-points at each 10^th^ percentile to aid interpretability. Parent study intervention status was not included as an exposure variable because school WASH facilities were available to the wider community and therefore inclusion of intervention status resulted in co-linearity with improved drinking water coverage. Furthermore, sensitivity analyses showed no association between parent study intervention status and pathogen prevalence, and the parent study showed no impact on the prevalence of diarrhea among beneficiaries of the intervention [44].

To examine the odds of enteric infection across age groups, we categorized each subject as a child <5 years old (CU5); school-aged child (SAC), defined as a child enrolled in primary school (grades 1–5); or adult. Socioeconomic status was determined through a series of questions and observations about household construction materials (roof, floor, and walls), ownership of a mobile phone, and presence of electricity. These variables were chosen based on those used in the Demographic and Health Surveys for measures of wealth in Lao PDR [50], and we used principal component analysis methods to derive one single wealth metric from all of the wealth assets combined [51]. The number of household members was defined as all people currently living in the household full time at the time of the survey.

*E*. *coli* pathotypes were classified according to the following gene targets: EAEC (*aatA* and/or *aaiC*), EHEC (*eae* with *stx1* and/or *stx2*, and without *bfpA*), typical EPEC (*bfpA* with or without *eae*), atypical EPEC (*eae* without *bfpA*, *stx1*, or *stx2*), ETEC (*eltB* for heat-labile toxin [LT] and *estA* with or without *eltB* for heat-stable toxin [ST]) [25].

### Statistical analysis

All data were stored and managed using Stata Statistical Software: Release 15 (StataCorpLP, College Station, TX, USA). Statistical analyses were conducted using Stata and R version 3.5.2 (Vienna, Austria).

We calculated odds ratios (ORs) and 95% confidence intervals (CIs) for each primary and secondary outcome using mixed effects logistic regression models, with random intercepts at the village and household levels to account for clustering. For the primary outcomes, we tested for effect modification between household WASH covariates and sex and between household WASH covariates and age group using an interaction term between relevant covariates in the fully adjusted models. There was no evidence of effect modification and the interaction term was not included in the models.

To measure intra-household infection concordance, we conducted an association screening analysis [52]. For each enteropathogen, we created an absence/presence matrix with households in rows and hosts (CU5, SAC, Adult) in columns. The association screening analysis creates permutation-based 95% confidence bounds around the expected frequency of infection for each possible combination of household members given the overall pathogen prevalence within each population. Intra-household infection/host combinations with frequencies observed above or below these bounds represent combinations that occur more or less frequently, respectively, than would be expected by chance [52]. We define household triad infection concordance as all three household members having the same infection, and partial infection concordance as two household members (CU5 and adult, CU5 and SAC, adult and SAC) having the same infection. This analysis was possible only when the pathogen was prevalent in at least one subject from each age group. Subjects were included in the analysis only when pathogen data was available for the complete household triad.

To estimate the association between village- and household-level clustering and odds of infection, we calculated the median odds ratio (MOR) of the random intercepts. The MOR translates area-level variance to the OR scale, and can be interpreted as the median increased odds of infection that one would have by moving to another area (village or household) with higher odds of infection [53]. In other words, the MOR represents the extent to which an individual’s odds of infection are determined by its village or household, after adjusting for other measured covariates [53, 54].

We also examined the intraclass correlation coefficient (ICC), which estimates the proportion of observed variation in the outcome due to clustering after accounting for covariate effects. Because we used logistic regression, we employed the latent variable method, which converts both the individual- and area-level components of the variance to the logistic scale prior to computing the ICC [53]. ICC scores range from 0 to 1; a low value indicates that village/household residual variations are relatively independent and suggests that unmeasured village/household level factors are not relevant to understanding differences in the outcome, whereas a value closer to 1 indicates that unmeasured village/household-level factors are strongly associated with the outcome [55].

All analyses were evaluated for statistical significance using a two-sided *α* = 0.05.

## Results

We collected a total of 2,269 fecal samples from participants in 1,159 households. Of these, all three subjects in the household (CU5, SAC, and adult) returned their stool sample on the same day in 297 households (891 subjects) and thus were eligible for inclusion in the study. There were no meaningful differences in measured exposures between households included in the study and those that were not (**S2 Table)**. One sample was excluded due to insufficient amount for nucleic acid extraction, so samples from 890 participants were included in the analysis. Based on field and laboratory extraction blanks, we suspected contamination by one or more target pathogen of 66 samples in the field (EPEC = 1, rotavirus = 11, *Shigella*/EIEC = 21, STEC *stx2* = 33, EAEC = 40, *C*. *difficile* = 1, *A*. *lumbricoides* = 1) and 78 samples in the laboratory (rotavirus = 64, astrovirus = 3, *C*. *jejuni/C*. *coli* = 8); these samples were excluded from taxa- and pathogen-specific analyses.

### Description of study population, WASH access, and pathogen prevalence

Household and community-level WASH factors are described in **Table 1**. One hundred and fifty (50.5%) of CU5 were female, 143 (48.2%) of SAC were female, and all adult participants were female.

**Table 1:** Description of study population, Saravane Province, Lao PDR, 2017.

	Total (*n* = 297 households)
**Household-level characteristics**	
Household population size, median (IQR)	7 (3.0)
Toilet type, n (%)	
Pit latrine without slab	18 (6.1%)
Pit latrine with slab	15 (5.1%)
Pour flush	49 (16.5%)
No toilet	215 (72.4%)
Improved toilet^1^, n (%)	67 (22.6%)
Water source, n (%)	
Surface water	39 (13.1%)
Unprotected well	60 (20.2%)
Unprotected spring	18 (6.1%)
Protected well	3 (1.0%)
Protected spring	3 (1.0%)
Borehole	77 (25.9%)
Rainwater	2 (0.7%)
Piped to house	11 (3.7%)
Piped to yard/compound	6 (2.0%)
Public tap/standpipe	38 (12.8%)
Bottled water	40 (13.5%)
Improved drinking water source^1^, n (%)	140 (47.2%)
Basic handwashing facility^1^, n (%)	100 (33.7%)
Animal ownership, n (%)	282 (94.9%)
**Village-level characteristics**	
Improved sanitation^1^ coverage, median % (IQR)	8.3% (41.7%)
Improved drinking water source^1^ coverage, median % (IQR)	37.5% (79.2%)
Basic handwashing facility^1^ coverage, median % (IQR)	33.3% (25.0%)

IQR = interquartile range

^1^Defined according to according to WHO/UNICEF Joint Monitoring Programme standards [49]

Pathogen prevalence by age group is described in **Table 2**. One or more enteropathogens were identified in 875 (98.3%) of the subjects. The median (IQR) number of enteropathogen infections per person was 4.0 (3.0), with no variation by age group. Bacterial infections were the most prevalent, with 85.2% of subjects having at least one bacterial infection, followed by protozoal infections (74.9% of subjects), STH infections (63.9% of subjects), and viral infections (34.6% of subjects). The most common enteropathogens detected were Giardia (70.9%), hookworm (48.4%), EAEC (47.8%), ETEC (36.9%), and EPEC (35.2%).

**Table 2:** Prevalence of enteropathogens, stratified by age group and ordered from most to least prevalent, Saravane Province, Lao PDR, 2017.

	CU5* (*n* = 297)	SAC* (*n* = 297)	Adult (*n* = 296)	Total *(n* = 890)
Any enteropathogen	294 (99.0%)	292 (98.3%)	289 (97.6%)	875 (98.3%)
Median (IQR) enteropathogens	4 (3.0)	4 (3.0)	4 (3.0)	4 (3.0)
Any bacteria^1^	241 (86.1%)	230 (82.7%)	239 (86.9%)	710 (85.2%)
Any protozoa	249 (83.8%)	231 (77.8%)	187 (63.2%)	667 (74.9%)
Any STH^1^^*^	163 (55.1%)	197 (66.3%)	208 (70.3%)	568 (63.9%)
Any virus^1^	104 (37.6%)	90 (32.7%)	92 (33.5%)	286 (34.6%)
*Giardia intestinalis*	238 (80.1%)	221 (74.4%)	172 (58.1%)	631 (70.9%)
Hookworm	110 (37.0%)	158 (53.2%)	163 (55.1%)	431 (48.4%)
EAEC^1^^*^	128 (44.9%)	129 (45.1%)	149 (53.4%)	406 (47.8%)
ETEC^*^	107 (36.0%)	89 (30.0%)	132 (44.6%)	328 (36.9%)
EPEC^1^^*^	109 (37.7%)	103 (35.9%)	89 (31.8%)	301 (35.2%)
*Aeromonas* spp.	64 (21.5%)	80 (26.9%)	111 (37.5%)	255 (28.7%)
Rotavirus^1^	72 (26.0%)	67 (24.4%)	72 (26.2%)	211 (25.5%)
*Campylobacter jejuni*^1^	82 (27.8%)	71 (24.2%)	43 (14.6%)	196 (22.2%)
*Strongyloides stercoralis*	43 (14.5%)	58 (19.5%)	84 (28.4%)	185 (20.8%)
*Shigella*/EIEC^1^^*^	47 (16.4%)	48 (16.4%)	54 (18.7%)	149 (17.1%)
*Trichuris trichiura*	49 (16.5%)	55 (18.5%)	42 (14.2%)	146 (16.4%)
*Cryptosporidium* spp.	56 (18.9%)	42 (14.1%)	42 (14.2%)	140 (15.7%)
EHEC^*^	23 (7.7%)	35 (11.8%)	49 (16.6%)	107 (12.0%)
*Ascaris lumbricoides*^1^	29 (9.8%)	28 (9.4%)	24 (8.1%)	81 (9.1%)
Norovirus GII	24 (8.1%)	21 (7.1%)	24 (8.1%)	69 (7.8%)
*Salmonella enterica*	10 (3.4%)	9 (3.0%)	23 (7.8%)	42 (4.7%)
Astrovirus	11 (3.7%)	8 (2.7%)	4 (1.4%)	23 (2.6%)
Sapovirus	12 (4.0%)	4 (1.3%)	1 (0.3%)	17 (1.9%)
*Clostridium difficile*^1^	4 (1.3%)	3 (1.0%)	3 (1.0%)	10 (1.1%)
Norovirus GI	1 (0.3%)	4 (1.3%)	3 (1.0%)	8 (0.9%)
Adenovirus 4041	0 (0.0%)	4 (1.3%)	2 (0.7%)	6 (0.7%)
*Cryptosporidium hominus*	0 (0.0%)	1 (0.3%)	0 (0.0%)	1 (0.1%)
*Entamoeba histolytica*	1 (0.3%)	0 (0.0%)	0 (0.0%)	1 (0.1%)
*Cryptosporidium parvum*	0 (0.0%)	0 (0.0%)	0 (0.0%)	0 (0.0%)


*CU5 = child under-5, SAC = school-aged child, STH = soil-transmitted helminth, EAEC = enteroaggregative *Escherichia coli*, EHEC = enterohemorrhagic *E*. *coli*, EPEC = enteropathogenic *E*. *coli*, ETEC = enterotoxigenic *E*. *coli*, EIEC = enteroinvasive *E*. *coli*

^1^number of samples missing due to suspected field or laboratory contamination: virus = 63, bacteria = 57, STH = 1, EAEC = 40, EPEC = 34, rotavirus = 63, *C*. *jejuni/C*. *coli* = 8, *Shigella*/EIEC = 11, *A*. *lumbricoides* = 1, *C*. *difficile* = 1

### Associations between age and odds of enteropathogen infection

Unadjusted associations between covariates and enteropathogens are shown in **S3 Table**. Odds of enteropathogen infection differed by age group for protozoal and STH infections, but not for viral and bacterial infections (**Table 3**). Odds of protozoal infection decreased with age; compared to adults, protozoal infection was more likely among CU5 (OR = 3.12, 95% CI = 1.92, 5.07) and SAC (OR = 1.94, 95% CI = 1.22, 3.07). Odds of STH infection increased with age; compared to adults, STH infection was less likely among CU5 (OR = 0.40, 95% CI = 0.25, 0.64), but there was no difference between SAC and adults.

**Table 3:** Adjusted odds ratios and 95% confidence intervals of associations between demographic and WASH covariates and viral, bacterial, protozoal, and soil-transmitted helminth (STH) enteric infections, Saravane Province, Lao PDR, 2017.

	Virus^1^ (*n* = 827)	Bacteria^2^ (*n* = 833)	Protozoa^3^ (*n* = 890)	STH^4^ (*n* = 889)
Child <5 years (ref: adult)	1.15 (0.69, 1.91)	0.97 (0.53, 1.76)	***3*.*12 (1*.*92*, *5*.*07)***	***0*.*40 (0*.*25*, *0*.*64)***
School-aged child (ref: adult)	0.82 (0.49, 1.37)	0.72 (0.40, 1.30)	***1*.*94 (1*.*22*, *3*.*07)***	0.80 (0.50, 1.28)
Female (ref: male)	0.75 (0.46, 1.22)	1.12 (0.66, 1.90)	0.66 (0.41, 1.05)	1.07 (0.70, 1.62)
Socioeconomic status	0.88 (0.75, 1.04)	1.01 (0.86, 1.18)	1.06 (0.94, 1.20)	***0*.*85 (0*.*76*, *0*.*96)***
Household population size	0.94 (0.86, 1.04)	0.95 (0.88, 1.03)	1.02 (0.95, 1.10)	1.03 (0.96, 1.10)
Improved toilet	***2*.*79 (1*.*26*, *6*.*18)***	0.46 (0.21, 1.01)	0.85 (0.46, 1.57)	0.71 (0.40, 1.25)
Improved drinking water source	1.48 (0.76, 2.88)	0.64 (0.33, 1.25)	0.88 (0.52, 1.49)	1.01 (0.60, 1.70)
Basic handwashing facility	***0*.*44 (0*.*24*, *0*.*82)***	0.95 (0.54, 1.70)	0.76 (0.48, 1.21)	***0*.*54 (0*.*35*, *0*.*85)***
Household animal ownership	1.32 (0.37, 4.74)	1.34 (0.46, 3.89)	***3*.*59 (1*.*53*, *8*.*46)***	2.18 (0.87, 5.45)
Improved toilet coverage^5^	1.01 (0.85, 1.21)	1.08 (0.94, 1.24)	0.96 (0.86, 1.08)	***0*.*82 (0*.*73*, *0*.*93)***
Improved drinking water coverage^5^	0.97 (0.85, 1.11)	1.00 (0.90, 1.11)	0.93 (0.84, 1.01)	1.00 (0.91, 1.11)
Basic handwashing facility coverage^5^	0.94 (0.75, 1.18)	1.00 (0.85, 1.18)	0.95 (0.82, 1.10)	0.98 (0.84, 1.14)
Median Odds Ratio- Village^6^	3.89 (2.64, 6.69)	1.97 (1.48, 3.25)	2.07 (1.59, 3.10)	2.46 (1.89, 3.56)
Median Odds Ratio- Household^6^	3.10 (2.23, 4.94)	2.27 (1.58, 4.27)	1.96 (1.45, 3.39)	1.73 (1.28, 3.29)
ICC- Village	0.30	0.11	0.13	0.20
ICC- Household	0.21	0.16	0.11	0.07

All models include random intercepts at the village and household levels to account for clustering.

Bold italicized associations indicate statistical significance at p<0.05

^1^ Virus includes one or more of the following pathogens: astrovirus, adenovirus, norovirus GI, norovirus GII, rotavirus, or sapovirus.

^2^ Bacteria includes one or more of the following pathogens: *Aeromonas*, *C. difficile*, *C. jejuni*, EAEC, EHEC, EPEC (typical or atypical), LT- or ST-ETEC, *Shigella* spp./EIEC, or *Salmonella*.

^3^ Protozoa includes one or more of the following pathogens: non-hominus and non-parvum *Cryptosporidium* spp., *C. hominus*, *C. parvum*, *E. histolytica*, and *G. intestinalis*.

^4^ Soil-transmitted helminths (STH) includes one or more of the following helminths: hookworm (*N. americanus* and/or *A. duodenale*), *A. lumbricoides*, *T. trichiura*, or *S. stercoralis*.

^5^WASH covariate coverage is interpreted as the change in odds of infection per 10% increase in WASH covariate coverage at the village level

^6^Median odds ratio is interpreted as the median increased odds of infection that one would have if moving to another area (household or village) with higher odds of infection, after accounting for other covariates in the model.

**Fig 1:**
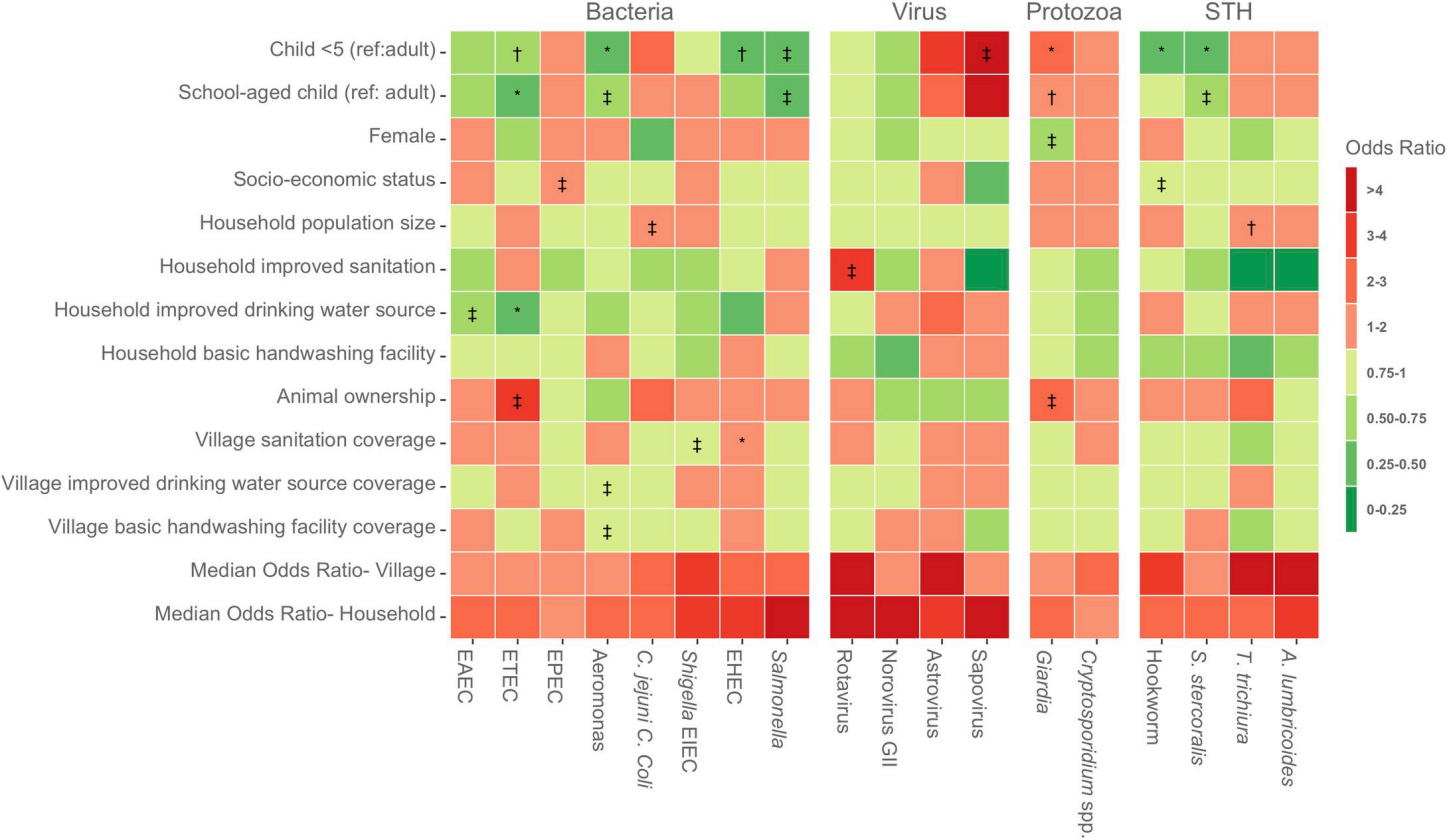
Heat map of adjusted odds ratios of associations between demographic and water, sanitation, and hygiene (WASH) covariates and enteropathogen infection. * indicates statistical significance at *p*<0.05, ^†^ indicates statistical significance at *p*<0.01, ^‡^ indicates statistical significance at *p*<0.001; *p*-values were not calculated for median odds ratios.

Similarly, pathogen-specific odds of infection differed by age group for some but not all pathogens (**Fig 1**). CU5 had higher odds of *Giardia*, *C*. *jejuni*, and sapovirus infection, but lower odds of ETEC, *Aeromonas*, EHEC, *Salmonella*, hookworm, and *S*. *stercoralis* infection, compared to adults. SAC had higher odds of *Giardia*, but lower odds of ETEC, *Aeromonas*, *Salmonella*, and *S*. *stercoralis* infection, compared to adults.

### Intra-household infection concordance

**Fig 2:**
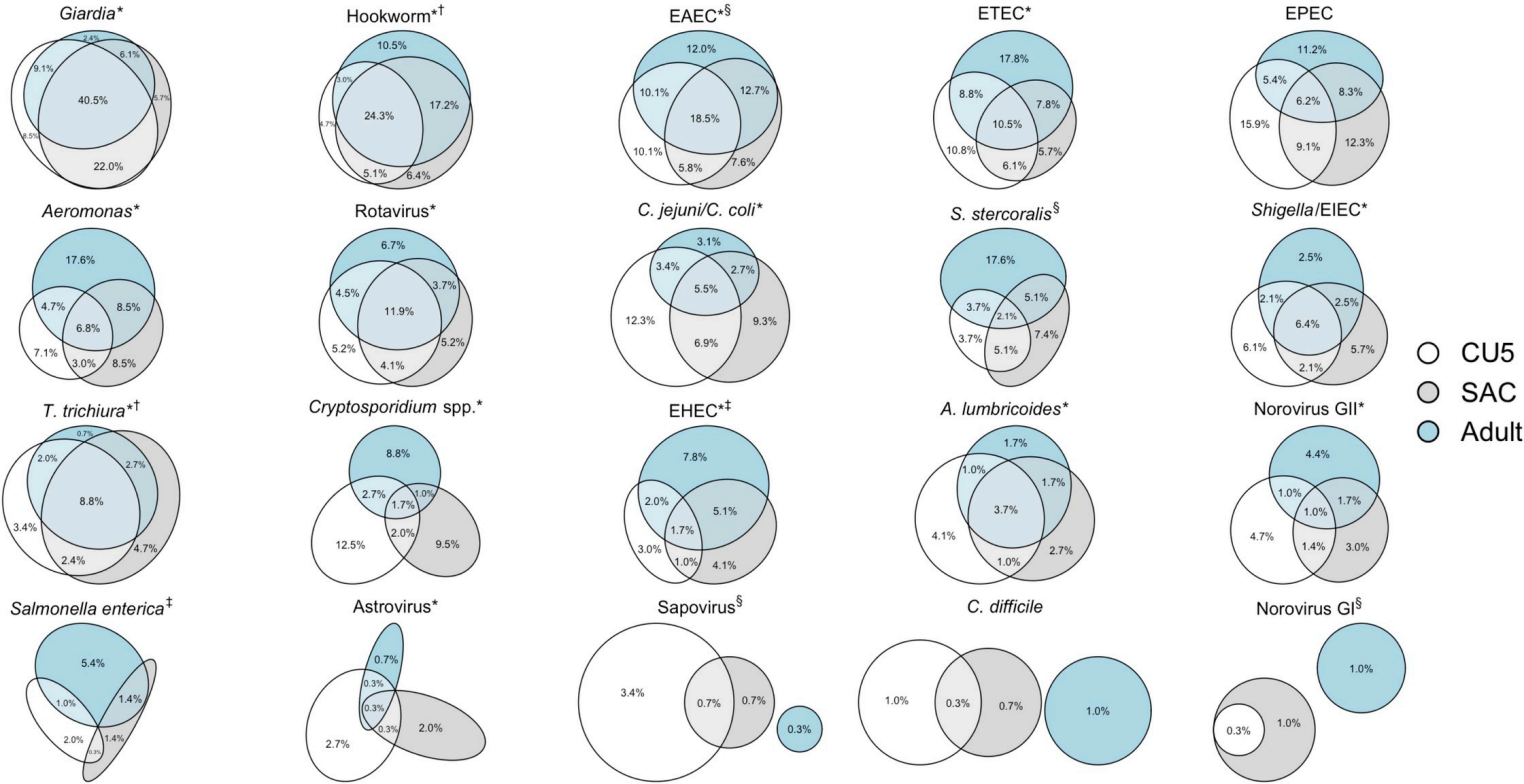
Intra-household infection concordance by enteropathogen, ordered from most to least prevalent enteropathogen in the population. Concordance of pathogen infection among children under 5 (CU5), school-aged children (SAC), and adults living in the same household and association screening analysis results. * indicates household triad infection concordance, *p*<0.05; ^†^ indicates infection concordance among the adult & CU5, *p*<0.05; ^‡^ indicates infection concordance among the adult & SAC, *p*<0.05; § indicates infection concordance among the SAC & CU5, *p*<0.05.

Concordance of pathogen infection among CU5, SAC, and adults living in the same household is shown in **Fig 2**. Household triad infection concordance was statistically higher than expected by chance for two thirds of the pathogens: *Giardia* (40.5%, p = 0.03), hookworm (24.3%, *p*<0.001), EAEC (18.5%, *p*<0.001), ETEC (10.5%, *p*<0.001), rotavirus (11.9%, *p*<0.001), *T*. *trichiura* (8.8%, *p*<0.001), *Aeromonas* (6.8%, *p*<0.001), *Shigella* EIEC (6.4%, *p*<0.001), *C*. *jejuni* (5.5% *p*<0.001), *A*. *lumbricoides* (3.7%, *p*<0.001), *Cryptosporidium* spp. (1.7%, p = 0.02), EHEC (1.7%, p = 0.004), norovirus GII (1.0%, *p*<0.001), astrovirus (0.3%, *p* = 0.006). Concordance among the adult and CU5 household pair was statistically higher than expected only for hookworm (2.0%, *p*<0.001). Concordance among the adult and SAC household pair was statistically higher than expected for *Salmonella* (5.4%, *p* = 0.01) and EHEC (5.1%, *p* = 0.01). Concordance among the SAC and CU5 household pair were statistically higher than expected for EAEC (5.8%, *p =* 0.05), *S*. *stercoralis* (5.1%, *p* = 0.01), hookworm (5.1%, *p* = 0.01), and sapovirus (0.7%, *p* = 0.03).

### Bacteria

No WASH covariates were statistically associated with bacterial infection, though point estimates for household WASH access trended towards a protective association, while point estimates for community sanitation coverage and household animal ownership trended towards higher infection odds. The MOR-HH for bacterial infection (OR = 2.27, 95% CI = 1.58, 4.27) was higher than the MOR-V (OR = 1.97, 95% CI = 1.48, 3.25). After adjusting for model covariates, household-level clustering explained 16% (ICC = 0.16) of the remaining residual variance in odds of bacterial infection while village-level clustering explained 11% (ICC = 0.11).

### Viruses

Improved sanitation in the household was associated with higher odds of viral infection (OR = 2.79, 95% CI = 1.26, 6.18). A basic handwashing facility in the household was associated with lower odds of viral infection (OR = 0.44, 95% CI = 0.24, 0.82). Animal ownership was not associated with viral infection. The MOR-V for viral infection (MOR = 3.89, 95% CI = 2.64, 6.69) was higher than the MOR-HH (MOR = 3.10, 95% CI = 2.23, 4.94). After adjusting for model covariates, 30% of remaining residual variation in odds of viral infection (ICC = 0.30) was due to clustering at the village-level, and 21% was due to household-level clustering (ICC = 0.21).

### Protozoa

Household and community WASH covariates were not statistically associated with protozoal infection, though all point estimates trended towards a protective association. Animal ownership was associated with higher odds of protozoal infection (OR = 3.59, 95% CI = 1.53, 8.46). The MOR-V (MOR = 2.07, 95% CI = 1.59, 3.10) was similar to the MOR-HH (MOR = 1.96, 95% CI = 1.45, 3.39), and both were of greater relevance to odds of protozoal infection than were WASH covariates, but not age or animal ownership. After adjusting for model covariates, village-level clustering explained 13% of remaining residual variance in odds of protozoal infection (ICC = 0.13) while household-level clustering explained 11% (ICC = 0.11).

### STH

The presence of a basic handwashing facility in the household (OR = 0.54, 95% CI = 0.35, 0.85) and increasing improved sanitation coverage (OR = 0.82, 95% CI = 0.73, 0.93) were associated with lower odds of STH infection. Although not statistically associated with STH infection, point estimates for household-level improved toilet and basic handwashing facility coverage trended towards a protective association, while point estimates for household and village-level improved drinking water sources and animal ownership trended towards higher infection odds. The MOR-V for STH infection (MOR = 2.46, 95% CI = 1.89, 3.56) was higher than the MOR-HH (MOR = 1.73, 95% CI = 1.28, 3.29). Village-level clustering explained 20% of the remaining residual variance in odds of STH infection (ICC = 0.20) while household-level clustering explained 7% (ICC = 0.07).

## Discussion

In this study, we examined the leading pathogenic causes of enteric infections and household- and village-level risk factors for those infections across differently-aged study subjects living in the same households in rural Lao PDR. We detected a high prevalence of enteropathogens among our study population, with 98.3% of subjects harboring at least one enteropathogen infection. There was high concordance of infection within the household triad for many enteropathogen species, indicating the importance of intra-household transmission, even among school-aged children and adults. Few household or village-level WASH covariates we assessed were statistically associated with odds of infection at the taxa- or individual pathogen-level, though WASH access generally trended towards lower odds of infection.

At the taxa-level, we observed higher odds of infection among CU5 compared to other age groups only for protozoa, an association driven largely by *Giardia*, which is one of the first enteric pathogens to infect children [56], and the most common infection in our study population. We found no significant difference in odds of infection across age groups for half of the 18 pathogens in our species-specific analysis, including rotavirus, which is the leading cause of acute gastroenteritis in infants and young children in developing countries [57], the leading cause of death due to diarrhea among children <5 [58], and is typically considered a childhood illness [59]. One explanation for these associations is that intra-household transmission may be a key transmission pathway for enteropathogens. We found that concordance of pathogen infection among the whole household triad was statistically higher than expected for 14 of the 20 pathogens analyzed, and was more common than partial concordance of infection among specific household pairs (e.g. adult and CU5, adult and SAC, SAC and CU5). Additionally, household- and village-level clustering, as measured by the MOR, indicated substantial area-level variations relevant to understanding individual odds of infection. Together, these results suggest that the role of other household and village members in disease transmission should not be overlooked. More efforts to target all household members—including older children, adolescents, and adults—in etiological surveys of enteric illness and WASH interventions is warranted. Future research could further examine intra-household transmission patterns of enteropathogens.

Our measure of improved sanitation was largely representative of whether households reported using a toilet at all; an unimproved toilet was observed in only 5% of households and 75% of households reported open defecation by at least one household member. We observed that household-level improved sanitation was associated with higher odds of viral infection, while a household-level basic handwashing facility was associated with lower odds of viral infection. Compared to bacteria and protozoa, viruses have a lower infectious dose and a higher rate of shedding, sometimes long after resolution of symptoms. As a result, viral pathogens spread easily from person to person and via fomites [57, 60, 61]. Evidence suggests that improvements in sanitation alone are not sufficient to prevent enteric virus transmission [62], especially rotavirus which is highly infectious and extremely persistent in the environment [63]. Our results are consistent with research from India which reported an increased risk (though not significantly) of previous viral infection among urban households with toilets [64]. Additionally, a study among schools in rural Kenya reported higher hand contamination among schools that were provided improved toilets, but where inadequate hand hygiene was observed [65]. Hygiene of both hands and surfaces are critical to interrupting enteric virus transmission [62]. Our results substantiate evidence that without concurrent changes in hygiene, it is unlikely that sanitation alone will reduce incidence of enteric virus infections [59].

Our results are consistent with the established transmission pathways for STH via ingestion of eggs and contact with fecally contaminated food and soil [56]. We observed that a household basic handwashing facility was associated with 46% lower odds of STH infection. Having a toilet at the household level was not statistically associated with STH (though trended towards lower odds of infection), however, each 10% increase in community sanitation coverage was associated with 18% lower odds of STH infection. Current STH control strategies focus predominately on preventative chemotherapy (PC) of SACs [66], but re-infection frequently occurs quickly following treatment [67]. Thus, long-term control requires eliminating the environmental reservoir for STH through improvements in WASH, particularly sanitation [6]. Furthermore, household latrines will not prevent hookworm infection if open defecation still persists by some members of the community [30]. Our results support the limited evidence that both PC and WASH are necessary for sustained control or elimination of STH, as long as sanitation reaches a high level of uptake [68]. To our knowledge, there is no evidence on community thresholds of sanitation associated with STH, as has been done for diarrhea, trachoma, nutritional status, and infant mortality [31–39]. Such evidence would be of great benefit to the WASH and NTD sectors to influence policy on STH programming and coordination between sectors [69].

Although domestic animals are associated with increased pathogen diversity in the public domain [21], animal feces is often not taken into consideration in the design of household or community WASH interventions, which may partially explain the lack of effect observed in recent randomized trials [26, 27]. Our results support conclusions from recent reviews that exposure to animal feces is a risk factor for enteropathogen infection, and consequently on diarrhea, NTDs, and nutritional outcomes [26, 27, 70]. Many enteric bacteria, protozoa, and some STH can be transmitted by animal feces [26]. Zoonotic transmission of enteric viruses are rare, with the exception of rotavirus and Hepatitis E [26, 57]. Consistent with these pathways, we found that animal ownership was associated with higher odds of protozoal infection, and trended towards higher odds of bacterial and STH infection and lower odds of viral infection. Our results point to animal ownership as a possible risk factor for many enteric infections, which may or may not outweigh the potential benefit to increased socio-economic and nutritional status they may confer.

We found limited associations between measures of improved WASH access and enteric pathogen infection. Our results corroborate recent evidence from the SHINE trial showing no impact of WASH interventions (combined sanitation, water chlorination, and handwashing with soap) on enteric infections, also measured by TAC [15]. These results could indicate that the traditionally used measures of improved/unimproved WASH access, as defined by the WHO/UNICEF Joint Monitoring Programme [49], may not be nuanced enough to capture relevant WASH transmission pathways and behaviors. Additionally, these measures may not sufficiently account for other important transmission pathways such as flies, food contamination, and stored drinking water. Further, our multilevel analysis approach, which allowed us to estimate the residual variation between villages and households, highlighted the importance of contextual factors beyond our measured WASH parameters that influence one’s susceptibility to enteric infections. We found that the village-level MOR was higher than the household-level MOR for all taxa except bacteria, meaning that the individual probability of infection not explained by the current set of covariates was influenced more by village-level factors than by household-level factors. Recent evidence has demonstrated a substantial risk of enteric infection from the public domain by quantifying a diversity of enteropathogens in surface water, community water sources, and soil, including children’s play sites [21, 71]. Additionally, children’s exposure to enteric pathogens in their neighborhood may have spatial dimensions; the more area they have contact with in their neighborhood, the greater their risk of multi-pathogen exposure and pathogen dose [41]. These results suggest that interventions addressing both household- and community-level exposures may be necessary, particularly in places where enteric viruses, protozoa, and STH are the predominant etiologies of enteric illness.

### Strengths and limitations

This study has several strengths. First, measuring enteropathogen prevalence among adults, children, and infants residing in the same household allowed us to quantify associations between village- and household-level clustering and enteropathogen infection. Second, diarrhea is considered a disease of importance only for young children, despite evidence that morbidity is also high among older children, adolescents, and adults [72]. Our study is one of the few enteropathogen surveys to include older children and adults, and we observed high levels of enteropathogen infection across age groups. Third, participating villages and households were randomly selected. Fourth, we detected and quantified enteropathogens using qPCR, which provides accurate, quantitative molecular detection of multiple infection targets [47]; the multi-target detection capacity allowed us to examine 25 infectious pathogens, including a number of pathogens for which prevalence data in Lao PDR and the Southeast Asia region is scarce. For example, *S. stercoralis* is considered one of most neglected STHs among the NTDs [73], and there is limited evidence on the prevalence of *S*. *stercoralis* in Lao PDR [74]. We observed an overall *S*. *stercoralis* prevalence of 20.8%, the second highest among the STH. Additionally, we observed a substantial prevalence of *Aeromonas* (28.7%), which is common in soil but has also been linked to a number of intestinal and extraintestinal infections [75, 76]. Though *Aeromonas* has been implicated in outbreaks of diarrhea [76] it is often overlooked as an etiological agent of diarrhea [76].

Our study is subject to limitations. First, we do not have reliable diarrhea data. Detection of enteric pathogens in stool via molecular assays such as TAC can indicate asymptomatic or symptomatic infection, shedding due to recent exposure, or transient pathogen carriage of non-colonizing pathogens [77]. The criteria for distinguishing between low intensity infections, versus transient carriage are poorly understood. Nonetheless, the detection of pathogens in stool indicates a person’s exposure to the pathogen, regardless of infection or symptom status, and even subclinical infections may lead to detrimental long term sequalae such as environmental enteropathy, malnutrition, and growth stunting [78–80]. Additionally, fecal waste from individuals with asymptomatic infections still represents an exposure risk to others [81]. Second, we identified laboratory contamination in 144 samples. If contamination was suspected, the observation was dropped from the relevant taxa- or pathogen-specific model. We ran a sensitivity analysis between models where all contaminated observations were dropped, regardless of taxa or pathogen, and models where only relevant contaminated taxa/pathogen were dropped, and identified no significant differences between the models. Third, we were unable to measure direct exposure to animal feces so we relied on animal ownership as a proxy, as has been done in the majority of previous studies on animal feces exposure [27, 70]. Fourth, because of the exploratory nature of this analysis, we did not adjust for multiple comparisons despite having numerous hypothesis tests, thus increasing the risk for a Type I error. Additional research is needed to substantiate results. Fifth, low prevalence of some pathogens may have limited power to detect statistical associations. Odds ratios were used instead of risk ratios for the primary effect measure to facilitate the median odds ratio analysis. Given the high prevalence of many of our outcomes, estimates may be inflated. Last, villages were randomly selected from the school-hosting villages participating in the parent trial. Additionally, households were eligible for inclusion only if they had a school-aged child attending a school participating in the WASH HELPS trial, a child<5 years living in the household, *and* the household triad all returned their stool sample on the same day. Similarly, preference was given to female caregivers. Characteristics of these villages, households, and adults may be different in behaviors and exposures from those in the wider community, which may limit the generalizability of our findings.

## Conclusions

In our study area, enteropathogen infection was nearly universal, even among school-aged children and adults, and many species-specific infections were clustered within households. These important findings point to the need to consider transmission within the household, even among those whom are frequently considered lower priority household members less at risk of morbidities and mortality due to diarrhea. We observed that household- and village-level WASH access was generally associated with lower odds of enteric infection, but few WASH covariates were statistically associated with enteric infection at either the taxa- or individual pathogen-level. Transmission pathways varied by enteropathogen taxa, underscoring the challenges of addressing both acute and chronic infections using many of the existing WASH intervention approaches. Our results suggest that WASH access, as expected, is associated with lower enteric illness, but WASH access as currently defined does not reveal a measurably protective association with infection for many etiologies.

## Supporting information

S1 Checklist STROBE checklist. (DOCX)

S1 Table Primers and Probes for Custom TaqMan Array Card. (DOCX)

S2 Table Differences in household demographic and WASH access covariates by study inclusion status, Saravane Province, Lao PDR, 2017. (DOCX)

S3 Table Unadjusted odds ratios and 95% confidence intervals of associations between demographic and WASH covariates and viral, bacterial, protozoal, and soil-transmitted helminth (STH) enteric infections, Saravane Province, Lao PDR, 2017. (DOCX)

S1 Data Dataset. (XLSX)
