# Supplementary material for: Environmental and spatial determinants of enteric pathogen infection in rural Lao People’s Democratic Republic: A cross-sectional study
Source: PLoS Negl Trop Dis. 2020 Apr 8;14(4):e0008180. doi: 10.1371/journal.pntd.0008180 (PMC7170279; doi:10.1371/journal.pntd.0008180)
Supplement: S1 Checklist — (DOCX) [file pntd.0008180.s001.docx]

**S1 Checklist: STROBE Checklist**

|  | Item No. | Recommendation | Page  No. | Relevant text from manuscript |
| --- | --- | --- | --- | --- |
| **Title and abstract** | 1 | (*a*) Indicate the study’s design with a commonly used term in the title or the abstract | Title  1 | Environmental and spatial determinants of enteric pathogen infection in rural Lao People’s Democratic Republic: A cross-sectional study  We conducted a cross-sectional survey in 50 villages in Saravane Province, Lao People’s Democratic Republic. |
|  |  | (*b*) Provide in the abstract an informative and balanced summary of what was done and what was found | 1 | From 297 households, we collected 891 fecal samples from one child <5, one school-aged child, and one adult living in the same household, and collected survey and observational data on household demographics, WASH access, and animal ownership. Fecal samples were analyzed for 25 enteropathogens using a qRT-PCR assay. We observed near universal infection with at least one enteropathogen (98.3%). Few household or village-level WASH covariates were statistically associated with enteropathogen infection. Concordant household infection was higher than expected under the independence assumption for 14 of the 21 pathogens for which we had sufficient data, indicating strong household correlation for many infections. Median Odds Ratios (MORs), a measure of cluster-level (e.g. village and household) influence on an individual’s odds of infection, were elevated at the village level, particularly for viruses (MOR: 3.89; 95% Confidence Interval [CI]: 2.64, 6.69), protozoa (MOR: 2.07; 95% CI: 1.59, 3.10), and soil-transmitted helminths (MOR: 2.46; 95% CI: 1.89, 3.56), indicating strong village-level differences in individuals' odds of enteric infections. |
| Introduction | | | |  |
| Background/rationale | 2 | Explain the scientific background and rationale for the investigation being reported | 2-3 | Interventions to improve water, sanitation, and hygiene are associated with 33%, 25%, and 30% reductions in risk of childhood diarrhea, respectively [4], and improved WASH is associated with reductions in *Giardia* [5] and neglected tropical diseases (NTDs) such as soil-transmitted helminths (STHs), trachoma, and schistosomiasis [6-9]. Yet, several recent, high-profile impact evaluations have failed to demonstrate a significant reduction from WASH on stunting and diarrhea [10-14], STH infection [10, 13], and other enteric infections [15]. |
| Objectives | 3 | State specific objectives, including any prespecified hypotheses | 4 | The aims of this study were to 1. estimate the prevalence of enteropathogens among children <5, school-aged children, and adults and identify differences by age group; 2. model associations between WASH transmission pathways, including household- and community-level WASH access and exposure to animal feces, and enteropathogen infections at the taxa- and pathogen-level (taxa defined here as bacteria, virus, protozoa, or STH); and 3. quantify clustering of enteropathogen infections at the household- and village-level. |
| Methods | | | |  |
| Study design | 4 | Present key elements of study design early in the paper | 5-6 | We conducted a household survey to collect information on household demographics, asset and animal ownership, recent illness among household members, and WASH access and behaviors. We also conducted structured observations of WASH facilities when present.  During the household survey, we distributed three pre-labeled, resealable plastic bags, each containing a plastic spoon to collect stool samples from the pupil, the pupil’s parent/caregiver (preference was given to female parent/caregiver due to evidence of mothers’ hand contamination as an important vector for household disease transmission [46]), and the pupil’s sibling <5 years old (if multiple siblings, preference was given to youngest sibling). Participants were instructed to collect the first stool on the following morning. Stool samples were collected in the morning and transported with a cold chain to the field laboratory within two hours of collection. |
| Setting | 5 | Describe the setting, locations, and relevant dates, including periods of recruitment, exposure, follow-up, and data collection | 4-5  6 | This cross-sectional study was nested within the Water, Sanitation, and Hygiene for Health and Education in Laotian Primary Schools (WASH HELPS) study, a longitudinal cluster-randomized trial evaluating a comprehensive school-based water, sanitation, and hygiene (WASH) intervention in 100 schools in Saravane Province, Lao PDR between September 2014 and May 2017.  All data were collected between February-April 2017 (dry season), prior to annual school-based chemotherapy for STH. The time frame corresponded with the final round of data collection and conclusion of the WASH HELPS study [43, 44]. |
| Participants | 6 | (*a*) *Cohort study*—Give the eligibility criteria, and the sources and methods of selection of participants. Describe methods of follow-up  *Case-control study*—Give the eligibility criteria, and the sources and methods of case ascertainment and control selection. Give the rationale for the choice of cases and controls  *Cross-sectional study*—Give the eligibility criteria, and the sources and methods of selection of participants | 5  6 | We selected 50 of the 100 school-hosting villages participating in the WASH HELPS study using stratified random sampling based on district and WASH HELPS study intervention status. In each village, we randomly selected 25 households meeting two eligibility criteria: 1) having a child attending the primary school participating in the WASH HELPS study, and 2) having a child <5 years old living in the household.  Households were eligible for inclusion only if all three subjects in the household (adult, school-aged child, and child <5 years old, “household triad”) returned their stool sample on the same day. Including multiple subjects from the same household allowed us to quantify household-level clustering of infection and distinguish village-level effects from household-level effects. |
|  |  | (*b*) *Cohort study*—For matched studies, give matching criteria and number of exposed and unexposed  *Case-control study*—For matched studies, give matching criteria and the number of controls per case |  |  |
| Variables | 7 | Clearly define all outcomes, exposures, predictors, potential confounders, and effect modifiers. Give diagnostic criteria, if applicable | 8-9 | In the primary analysis, the infection outcome variables were defined by presence/absence of any viral, bacterial, protozoal, or STH gene marker. In the secondary analysis, the outcome variables were presence/absence of each individual pathogen.  The exposure variables included household-level improved drinking water source (reported), improved sanitation facility (observed), and basic handwashing facility (observed), all classified according to WHO/UNICEF Joint Monitoring Programme standards [49]; animal ownership, which was reported as owning any cows, goats, sheep, poultry (chickens or ducks), or pigs; and village-level prevalence of an improved drinking water source (“improved drinking water coverage”), an improved sanitation facility (“improved sanitation coverage”), and a basic handwashing facility ("basic handwashing facility coverage"). Village-level WASH prevalence was calculated by aggregating household-level WASH access variables of all households in the sub-study at the village-level (cluster), excluding each individual’s own household in order to better represent indirect exposure and to avoid forced correlation between household- and village-level covariates [31]. Village-level WASH prevalence was re-scaled with cut-points at each 10^th^ percentile to aid interpretability. Parent study intervention status was not included as an exposure variable because school WASH facilities were available to the wider community and therefore inclusion of intervention status resulted in co-linearity with improved drinking water coverage. Furthermore, sensitivity analyses showed no association between parent study intervention status and pathogen prevalence, and the parent study showed no impact on the prevalence of diarrhea among beneficiaries of the intervention [44].  To examine the odds of enteric infection across age groups, we categorized each subject as a child <5 years old (CU5); school-aged child (SAC), defined as a child enrolled in primary school (grades 1-5); or adult. Socioeconomic status was determined through a series of questions and observations about household construction materials (roof, floor, and walls), ownership of a mobile phone, and presence of electricity. These variables were chosen based on those used in the Demographic and Health Surveys for measures of wealth in Lao PDR [50], and we used principal component analysis methods to derive one single wealth metric from all of the wealth assets combined [51]. The number of household members was defined as all people currently living in the household full time at the time of the survey.  *E. coli* pathotypes were classified according to the following gene targets: EAEC (*aatA* and/or *aaiC*), EHEC (*eae* with *stx1* and/or *stx2*, and without *bfpA*), typical EPEC (*bfpA* with or without *eae*), atypical EPEC (*eae* without *bfpA*, *stx1*, or *stx2*), ETEC (*eltB* for heat-labile toxin [LT] and *estA* with or without *eltB* for heat-stable toxin [ST]) [25]. |
| Data sources/ measurement | 8* | For each variable of interest, give sources of data and details of methods of assessment (measurement). Describe comparability of assessment methods if there is more than one group | 5  7 | We conducted a household survey to collect information on household demographics, asset and animal ownership, recent illness among household members, and WASH access and behaviors. We also conducted structured observations of WASH facilities when present.  Total nucleic acid was extracted from samples using the ZymoBIOMICS DNA/RNA Mini Kit (Zymo Research, Irvine, CA, USA). One extraction blank was included per batch to exclude the possibility of false positives from contamination during extraction. Extractions were transported on dry ice to the University of Iowa and analyzed on a ViiA7 thermocycler (Thermo Fisher, Carlsbad, CA, USA) via a 40 cycle quantitative reverse transcription polymerase chain reaction (qRT-PCR) analysis using a custom TaqMan Array Card (TAC) (Thermo Fisher, Carlsblad, CA, USA) with compartmentalized, probe-based qPCR assays for 25 enteropathogens [47, 48]. TAC primer and probe sequences are listed in **Table S1**. TAC preparation was based on the protocol described by Liu et al., with the exception of including 0.3 µM BSA to reduce inhibition of nucleic acid amplification [47].  Two researchers manually read TAC data; a third researcher resolved conflicting results. Multicomponent plots were inspected for increases in fluorescence for the FAM-based gene-specific probe to validate true amplification of the complete gene target. No false positive signals were observed for any pathogen gene in 25 water-only qPCR controls within 40 cycles of amplification. The limit of detection for all assays using the TAC card have been experimentally determined to lie between 33 and 40 cycle thresholds, as described elsewhere. Thus, a sample was initially classified as positive for a pathogen if amplification was detected for one or both duplicates of a gene assay. Samples were ultimately considered positive only if the corresponding field and extraction blanks were negative, otherwise the data were considered invalid [48]. |
| Bias | 9 | Describe any efforts to address potential sources of bias | 7 | Two researchers manually read TAC data; a third researcher resolved conflicting results. |
| Study size | 10 | Explain how the study size was arrived at | 6 | Sample size was based on the maximum number of households we could include given the study budget. |

| Quantitative variables | 11 | Explain how quantitative variables were handled in the analyses. If applicable, describe which groupings were chosen and why | 8-9 | See #7 | |
| --- | --- | --- | --- | --- | --- |
| Statistical methods | 12 | (*a*) Describe all statistical methods, including those used to control for confounding | 9-11 | | We calculated odds ratios (ORs) and 95% confidence intervals (CIs) for each primary and secondary outcome using mixed effects logistic regression models, with random intercepts at the village and household levels to account for clustering. For the primary outcomes, we tested for effect modification between household WASH covariates and sex and between household WASH covariates and age group using an interaction term between relevant covariates in the fully adjusted models. There was no evidence of effect modification and the interaction term was not included in the models.  To measure intra-household infection concordance, we conducted an association screening analysis [52]. For each enteropathogen, we created an absence/presence matrix with households in rows and hosts (CU5, SAC, Adult) in columns. The association screening analysis creates permutation-based 95% confidence bounds around the expected frequency of infection for each possible combination of household members given the overall pathogen prevalence within each population. Intra-household infection/host combinations with frequencies observed above or below these bounds represent combinations that occur more or less frequently, respectively, than would be expected by chance [52]. We define household triad infection concordance as all three household members having the same infection, and partial infection concordance as two household members (CU5 and adult, CU5 and SAC, adult and SAC) having the same infection. This analysis was possible only when the pathogen was prevalent in at least one subject from each age group. Subjects were included in the analysis only when pathogen data was available for the complete household triad.  To estimate the association between village- and household-level clustering and odds of infection, we calculated the median odds ratio (MOR) of the random intercepts. The MOR translates area-level variance to the OR scale, and can be interpreted as the median increased odds of infection that one would have by moving to another area (village or household) with higher odds of infection [53]. In other words, the MOR represents the extent to which an individual’s odds of infection are determined by its village or household, after adjusting for other measured covariates [53, 54].  We also examined the intraclass correlation coefficient (ICC), which estimates the proportion of observed variation in the outcome due to clustering after accounting for covariate effects. Because we used logistic regression, we employed the latent variable method, which converts both the individual- and area-level components of the variance to the logistic scale prior to computing the ICC [53]. ICC scores range from 0 to 1; a low value indicates that village/household residual variations are relatively independent and suggests that unmeasured village/household level factors are not relevant to understanding differences in the outcome, whereas a value closer to 1 indicates that unmeasured village/household-level factors are strongly associated with the outcome [55].  All analyses were evaluated for statistical significance using a two-sided *α*=0.05. |
|  |  | (*b*) Describe any methods used to examine subgroups and interactions |  | | See (a) |
|  |  | (*c*) Explain how missing data were addressed | 11 | | One sample was excluded due to insufficient amount for nucleic acid extraction, so samples from 890 participants were included in the analysis. Based on field and laboratory extraction blanks, we suspected contamination by one or more target pathogen of 66 samples in the field (EPEC=1, rotavirus=11, *Shigella*/EIEC=21, STEC *stx2*=33, EAEC=40, *C. difficile*=1, *A. lumbricoides*=1) and 78 samples in the laboratory (rotavirus=64, astrovirus=3, *C. jejuni/C. coli*=8); these samples were excluded from taxa- and pathogen-specific analyses. |
|  |  | (*d*) *Cohort study*—If applicable, explain how loss to follow-up was addressed  *Case-control study*—If applicable, explain how matching of cases and controls was addressed  *Cross-sectional study*—If applicable, describe analytical methods taking account of sampling strategy |  | | See (a) |
|  |  | (*e*) Describe any sensitivity analyses |  | |  |
| Results | | | | | |
| Participants | 13* | (a) Report numbers of individuals at each stage of study—eg numbers potentially eligible, examined for eligibility, confirmed eligible, included in the study, completing follow-up, and analysed | 11 | | We collected a total of 2,269 fecal samples from the same number of participants. Of these, all three subjects in the household (CU5, SAC, and adult) returned their stool sample on the same day in 297 households (891 subjects) and thus were eligible for inclusion in the study. One sample was excluded due to insufficient amount for nucleic acid extraction, so samples from 890 participants were included in the analysis. |
|  |  | (b) Give reasons for non-participation at each stage |  | | See (a) |
|  |  | (c) Consider use of a flow diagram |  | |  |
| Descriptive data | 14* | (a) Give characteristics of study participants (eg demographic, clinical, social) and information on exposures and potential confounders | 11  12  13 | | We collected a total of 2,269 fecal samples from participants in 1,159 households. Of these, all three subjects in the household (CU5, SAC, and adult) returned their stool sample on the same day in 297 households (891 subjects) and thus were eligible for inclusion in the study. There were no meaningful differences in measured exposures between households included in the study and those that were not (**Table S2)**.  Table 1  Table 2 |
|  |  | (b) Indicate number of participants with missing data for each variable of interest |  | | Included in each table |
|  |  | (c) *Cohort study*—Summarise follow-up time (eg, average and total amount) |  | |  |
| Outcome data | 15* | *Cohort study*—Report numbers of outcome events or summary measures over time |  | |  |
|  |  | *Case-control study—*Report numbers in each exposure category, or summary measures of exposure |  | |  |
|  |  | *Cross-sectional study—*Report numbers of outcome events or summary measures | 13  13 | | Pathogen prevalence by age group is described in **Table 2**. One or more enteropathogens were identified in 875 (98.3%) of the subjects. The median (IQR) number of enteropathogen infections per person was 4.0 (3.0), with no variation by age group. Bacterial infections were the most prevalent, with 85.2% of subjects having at least one bacterial infection, followed by protozoal infections (74.9% of subjects), STH infections (69.3% of subjects), and viral infections (34.6% of subjects). The most common enteropathogens detected were Giardia (70.9%), hookworm (48.4%), EAEC (47.8%), ETEC (36.9%), and EPEC (35.2%).  Table 2 |
| Main results | 16 | (*a*) Give unadjusted estimates and, if applicable, confounder-adjusted estimates and their precision (eg, 95% confidence interval). Make clear which confounders were adjusted for and why they were included | 14-15 | | Table 3 |
|  |  | (*b*) Report category boundaries when continuous variables were categorized | 14-15 | | Table 3 |
|  |  | (*c*) If relevant, consider translating estimates of relative risk into absolute risk for a meaningful time period |  | |  |

Continued on next page

| Other analyses | 17 | Report other analyses done—eg analyses of subgroups and interactions, and sensitivity analyses | 16 | Concordance of pathogen infection among CU5, SAC, and adults living in the same household is shown in **Figure 2**. Household triad infection concordance was statistically higher than expected by chance for two thirds of the pathogens: Giardia (40.5%, p=0.03), hookworm (24.3%, *p*<0.001), EAEC (18.5%, *p*<0.001), ETEC (10.5%, *p*<0.001), rotavirus (11.9%, *p*<0.001), *T. trichiura* (8.8%, *p*<0.001), *Aeromonas* (6.8%, *p*<0.001), *Shigella* EIEC (6.4%, *p*<0.001), *C. jejuni* (5.5% *p*<0.001), *A. lumbricoides* (3.7%, *p*<0.001), *Cryptosporidium* spp. (1.7%, p=0.02), EHEC (1.7%, p=0.004), norovirus GII (1.0%, *p*<0.001), astrovirus (0.3%, *p*=0.006). Concordance among the adult and CU5 household pair was statistically higher than expected only for hookworm (2.0%, *p*<0.001). Concordance among the adult and SAC household pair was statistically higher than expected for *Salmonella* (5.4%, *p*=0.01) and EHEC (5.1%, *p*=0.01). Concordance among the SAC and CU5 household pair were statistically higher than expected for EAEC (5.8%, *p=*0.05), *S. stercoralis* (5.1%, *p*=0.01), hookworm (5.1%, *p*=0.01), and sapovirus (0.7%, *p*=0.03). |
| --- | --- | --- | --- | --- |
| Discussion | | | | |
| Key results | 18 | Summarise key results with reference to study objectives | 18 | We detected a high prevalence of enteropathogens among our study population, with 98.3% of subjects harboring at least one enteropathogen infection. There was high concordance of infection within the household triad for many enteropathogen species, indicating the importance of intra-household transmission, even among school-aged children and adults. Few household or village-level WASH covariates we assessed were statistically associated with odds of infection at the taxa- or individual pathogen-level, though WASH access generally trended towards lower odds of infection. |
| Limitations | 19 | Discuss limitations of the study, taking into account sources of potential bias or imprecision. Discuss both direction and magnitude of any potential bias | 23-24 | Our study is subject to limitations. First, we do not have reliable diarrhea data. Detection of enteric pathogens in stool via molecular assays such as TAC can indicate asymptomatic or symptomatic infection, shedding due to recent exposure, or transient pathogen carriage of non-colonizing pathogens [77]. The criteria for distinguishing between low intensity infections, versus transient carriage are poorly understood. Nonetheless, the detection of pathogens in stool indicates a person’s exposure to the pathogen, regardless of infection or symptom status, and even subclinical infections may lead to detrimental long term sequalae such as environmental enteropathy, malnutrition, and growth stunting [78-80]. Additionally, fecal waste from individuals with asymptomatic infections still represents an exposure risk to others [81]. Second, we identified laboratory contamination in 144 samples. If contamination was suspected, the observation was dropped from the relevant taxa- or pathogen-specific model. We ran a sensitivity analysis between models where all contaminated observations were dropped, regardless of taxa or pathogen, and models where only relevant contaminated taxa/pathogen were dropped, and identified no significant differences between the models. Third, we were unable to measure direct exposure to animal feces so we relied on animal ownership as a proxy, as has been done in the majority of previous studies on animal feces exposure [27, 70]. Fourth, because of the exploratory nature of this analysis, we did not adjust for multiple comparisons despite having numerous hypothesis tests, thus increasing the risk for a Type I error. Additional research is needed to substantiate results. Fifth, low prevalence of some pathogens may have limited power to detect statistical associations. Odds ratios were used instead of risk ratios for the primary effect measure to facilitate the Median Odds Ratio analysis. Given the high prevalence of many of our outcomes, estimates may be inflated. Last, villages were randomly selected from the school-hosting villages participating in the parent trial. Additionally, households were eligible for inclusion only if they had a school-aged child attending a school participating in the WASH HELPS trial, a child<5 years living in the household, *and* the household triad all returned their stool sample on the same day. Similarly, preference was given to female caregivers. Characteristics of these villages, households, and adults may be different in behaviors and exposures from those in the wider community, which may limit the generalizability of our findings. |
| Interpretation | 20 | Give a cautious overall interpretation of results considering objectives, limitations, multiplicity of analyses, results from similar studies, and other relevant evidence | 24-25 | In our study area, enteropathogen infection was nearly universal, even among school-age children and adults, and many species-specific infections were clustered within households. These important findings point to the need to consider transmission within the household, even among those whom are frequently considered lower priority household members less at risk of morbidities and mortality due to diarrhea. We observed that household- and village-level WASH access was generally associated with lower odds of enteric infection, but few WASH covariates were statistically associated with enteric infection at either the taxa- or individual pathogen-level. Transmission pathways varied by enteropathogen taxa, underscoring the challenges of addressing both acute and chronic infections using many of the existing WASH intervention approaches. Our results suggest that WASH access, as expected, is associated with lower enteric illness, but WASH access as currently defined does not reveal a measurably protective association with infection for many etiologies. |
| Generalisability | 21 | Discuss the generalisability (external validity) of the study results | 24 | Villages were randomly selected from the school-hosting villages participating in the parent trial. Additionally, households were eligible for inclusion only if they had a school-aged child attending a school participating in the WASH HELPS trial, a child<5 years living in the household, *and* the household triad all returned their stool sample on the same day. Similarly, preference was given to female caregivers. Characteristics of these villages, households, and adults may be different in behaviors and exposures from those in the wider community, which may limit the generalizability of our findings. |
| Other information | |  | | |
| Funding | 22 | Give the source of funding and the role of the funders for the present study and, if applicable, for the original study on which the present article is based | Included in submission metadata | This study was funded by the Johnson and Johnson Foundation. ANC was supported by National Institutes of Health grant 5T32ES012870-12. The funders had no role in study design, data collection and analysis, decision to publish, or preparation of the manuscript |
