# Supplementary material for: Environmental and spatial determinants of enteric pathogen infection in rural Lao People’s Democratic Republic: A cross-sectional study
Source: PLoS Negl Trop Dis. 2020 Apr 8;14(4):e0008180. doi: 10.1371/journal.pntd.0008180 (PMC7170279; doi:10.1371/journal.pntd.0008180)
Supplement: S1 Table — (DOCX) [file pntd.0008180.s002.docx]

Table S1. Primers and Probes for Custom TaqMan Array Card.

| **Assay ID** | **Pathogen** | **Gene Target** | **Forward Primer** | **Reverse Primer** | **Probe Sequence** | **Ref.** |
| --- | --- | --- | --- | --- | --- | --- |
| APMFXDE | Adenovirus 40-41 | Fiber Gene | AACTTTCTCTCTTAATAGACGCC | AGGGGGCTAGAAAACAAAA | CTGACACGGGCACTCT | [1] |
| APNKRXC | EAEC *aaiC* | EAEC *aaiC* | ATTGTCCTCAGGCATTTCAC | ACGACACCCCTGATAAACAA | TAGTGCATACTCATCATTTAAG | [2] |
| APPRKG9 | EAEC *aatA* | EAEC *aatA* | CTGGCGAAAGACTGTATCAT | TTTTGCTTCATAAGCCGATAGA | TGGTTCTCATCTATTACAGACAGC | [2] |
| APRWE26 | EPEC *eae* | EPEC *eae* | CATTGATCAGGATTTTTCTGGTGATA | CTCATGCGGAAATAGCCGTTA | ATACTGGCGAGACTATTTCAA | [2] |
| APTZ9M3 | EPEC *bfpA* | EPEC *bfpA* | TGGTGCTTGCGCTTGCT | CGTTGCGCTCATTACTTCTG | CAGTCTGCGTCTGATTCCAA | [2] |
| APU627Z | ETEC LT | ETEC LT | TTCCCACCGGATCACCAA | CAACCTTGTGGTGCATGATGA | CTTGGAGAGAAGAACCCT | [2] |
| CCU002 | ETEC STh STp | STh STp | GCTAAACCAGYAGRGTCTTCAAAA TGAATCACTTGACTCTTCAAAA | CCCGGTACARGCAGGATTACAACA TGAATCACTTGACTCTTCAAAA | TGGTCCTGAAAGCATGAA TGAACAACACATTTTACTGCT | [2] |
| CCU001L | STEC *stx1* | STEC *stx1* | ACTTCTCGACTGCAAAGACGTATG | ACAAATTATCCCCTGWGCCACTATC | CTCTGCAATAGGTACTCCA | [2] |
| APXGRDV | STEC *stx2* | STEC *stx2* | CCACATCGGTGTCTGTTATTAACC | GGTCAAAACGCGCCTGATAG | TTGCTGTGGATATACGAGG | [2] |
| APYMJXT | *C. jejuni C. Coli* | *cadF* | CTGCTAAACCATAGAAATAAAATTTCTCAC | CTTTGAAGGTAATTTAGATATGGATAATCG | CATTTTGACGATTTTTGGCTTGA | [2] |
| APZTEHP | *C. difficile* | *tcdB* | GGTATTACCTAATGCTCCAAATAG | TTTGTGCCATCATTTTCTAAGC | CCTGGTGTCCATCCTGTTTC | [2] |
| AP2W73M | *Salmonella*  *enteritidis* | *ttr* | CTCACCAGGAGATTACAACATGG | AGCTCAGACCAAAAGTGACCATC | CACCGACGGCGAGACCGACTTT | [1] |
| AP322NJ | *Shigella* spp./EIEC | *ipaH* | CCTTTTCCGCGTTCCTTGA | CGGAATCCGGAGGTATTGC | CGCCTTTCCGATACCGTCTCTGCA | [2] |
| AP47V9G | *Cryptosporidium* | 18s rRNA | GGGTTGTATTTATTAGATAAAGAACCA | AGGCCAATACCCTACCGTCT | TGACATATCATTCAAGTTTCTGAC | [2] |
| AP7DPUE | *C. hominus* | LIB13 | TCCTTGAAATGAATATTTGTGACTCG | AAATGTGGTAGTTGCGGTTGAAA | CTTACTTCGTGGCGGCGT | [1] |
| AP9HJEC | *C. parvum* | LIB13 | TCCTTGAAATGAATATTTGTGACTCG | TTAATGTGGTAGTTGCGGTTGAAC | TATCTCTTCGTAGCGGCGTA | [1] |
| APAAAR2 | *E. histolytica* | 18s rRNA | ATTGTCGTGGCATCCTAACTCA | GCGGACGGCTCATTATAACA | TCATTGAATGAATTGGCCATTT | [2] |
| APCE4CY | *Giardia* | 18s rRNA | GACGGCTCAGGACAACGGTT | TTGCCAGCGGTGTCCG | CCCGCGGCGGTCCCTGCTAG | [2] |
| APDJXWW | *A. duodenale* | ITS2 | GAATGACAGCAAACTCGTTGTTG | ATACTAGCCACTGCCGAAACGT | ATCGTTTACCGACTTTAG | [1] |
| APEPTGU | *A. lumbricoides* | ITS1 | GCCACATAGTAAATTGCACACAAAT | GCCTTTCTAACAAGCCCAACAT | TTGGCGGACAATTGCATGCGAT | [1] |
| APFVK2R | *N. americanus* | ITS2 | CTGTTTGTCGAACGGTACTTGC | ATAACAGCGTGCACATGTTGC | CTGTACTACGCATTGTATAC | [1] |
| APGZFMN | *S. stercoralis* | Dispersed repetitive sequence | TCCAGAAAAGTCTTCACTCTCCAG | TGCGTTAGAATTTAGATATTATTGTTGCT | TCAGCTCCAGTTGAACAACAGCCTCCAA | [1] |
| APH497K | *T. trichiura* | 18s rRNA | TTGAAACGACTTGCTCATCAACTT | CTGATTCTCCGTTAACCGTTGTC | CGATGGTACGCTACGTGCTTACCATGG | [2] |
| APKA3TH | MS2 | MS2g1 | TGGCACTACCCCTCTCCGTATTCAC | GTACGGGCGACCCCACGATGAC | CACATCGATAGATCAAGGTGCCTACAAGC | [2] |
| CCU001L | Rotavirus | NSP3 | ACCATCTWCACRTRACCCTCTATGAG | GGTCACATAACGCCCCTATAGC | AGTTAAAAGCTAACACTGTCAAA | [2] |
| CCU001L | *Aeromonas* | Aerolysin | TYCGYTACCAGTGGGACAAG | CCRGCAAACTGGCTCTCG | CAGTTCCAGTCCCACCACTT | [1] |
| APMFXDF | Astrovirus | Capsid | CAGTTGCTTGCTGCGTTCA | CTTGCTAGCCATCACACTTCT | CACAGAAGAGCAACTCCATCGC | [2] |
| CCU001L | Norovirus GI | ORF 1-2 | CGYTGGATGCGNTTYCATGA | CTTAGACGCCATCATCATTYAC | TGGACAGGAGATCGC | [1] |
| CCU001L | Norovirus GII | ORF 1-2 | CARGARBCNATGTTYAGR TGGATGAG | TCGACGCCATCTTCATTCACA | TGGGAGGGCGATCGCAATCT | [2] |
| CCU002 | Sapovirus | RdRP | GAYCAGGCTCTCGCYACCTAC TTTGAACAAGCTGTGGCATGCTAC | CCCTCCATYTCAAACACTA | CYTGGTTCATAGGTGGTRCAG CAGCTGGTACATTGGTGGCAC | [2] |
| APNKRXD | PhHV | gB | GGGCGAATCACAGATTGAATC | GCGGTTCCAAACGTACCAA | TATGTGTCCGCCACCATCT | [2] |
| APPRKHA | *Enterococcus faecalis* | *ent* | GAGAAATTCCAAACGAACTTG | CAGTGCTCTACCTCCATCATT | TGGTTCTCTCCGAAATAGCTTTAGGGCTA | [3] |
| APRWE27 | EHEC *E. coli* 0157 | *rdbE* | TTTCACACTTATTGGATGGTCTCAA | CGATGAGTTTATCTGCAAGGTGAT | CTCTCTTTCCTCTGCGGTCCT | [1] |

**References**

1. Liu J, Gratz J, Amour C, Nshama R, Walongo T, Maro A, et al. Optimization of quantitative PCR methods for enteropathogen detection. PLoS One. 2016;11:e0158199.

2. Liu J, Gratz J, Amour C, Kibiki G, Becker S, Janaki L, et al. A laboratory-developed TaqMan Array Card for simultaneous detection of 19 enteropathogens. J Clin Microbiol. 2013;51:472–80.

3. Ludwig W, Schleifer KH. How quantitative is quantitative PCR with respect to cell counts? Syst Appl Microbiol. 2000;23:556-62.
