# Supplementary material for: Environmental and spatial determinants of enteric pathogen infection in rural Lao People’s Democratic Republic: A cross-sectional study
Source: PLoS Negl Trop Dis. 2020 Apr 8;14(4):e0008180. doi: 10.1371/journal.pntd.0008180 (PMC7170279; doi:10.1371/journal.pntd.0008180)
Supplement: S2 Table — (DOCX) [file pntd.0008180.s003.docx]

| Table S2. Differences in household demographic and WASH access covariates by study inclusion status, Saravane Province, Lao PDR, 2017 | | | |
| --- | --- | --- | --- |
|  | **Included**  (*n*=297) | **Excluded**  (n=888) | ***p^1^*** |
| **Household-level characteristics** | |  |  |
| Household population size, median (IQR) | 7.0 (3.0) | 6.0 (3.0) | 0.06 |
| Improved toilet^1^, n (%) | 67 (22.6%) | 174 (19.6%) | 0.58 |
| Improved drinking water source^1^, n (%) | 140 (47.2%) | 461 (53.5%) | 0.16 |
| Basic handwashing facility^1^, n (%) | 100 (33.7%) | 226 (25.5%) | 0.21 |
| Animal ownership, n (%) | 282 (94.9%) | 802 (90.3%) | 0.23 |
| **Village-level characteristics** | |  |  |
| Improved sanitation^1^ coverage, median % (IQR) | 8.3% (41.7%) | 12.5% (33.3%) | 0.47 |
| Improved drinking water source^1^ coverage, median % (IQR) | 37.5% (79.2%) | 50.0% (66.7%) | 0.13 |
| Basic handwashing facility^1^ coverage, median % (IQR) | 33.3% (25.0%) | 25.0% (33.3%) | 0.41 |
| IQR=interquartile range ^1^*p*-value based on mixed effects logistic regression models for categorical outcomes (improved toilet, improved drinking water, basic handwashing facility, animal ownership) and linear regression models for continuous outcomes (household population size, improved sanitation coverage, improved drinking water source coverage, and basic handwashing facility coverage). All models include random intercepts at the village level to account for clustering. ^2^Defined according to according to WHO/UNICEF Joint Monitoring Programme standards [55] | | | |
