# Supplementary material for: Environmental and spatial determinants of enteric pathogen infection in rural Lao People’s Democratic Republic: A cross-sectional study
Source: PLoS Negl Trop Dis. 2020 Apr 8;14(4):e0008180. doi: 10.1371/journal.pntd.0008180 (PMC7170279; doi:10.1371/journal.pntd.0008180)
Supplement: S3 Table — (DOCX) [file pntd.0008180.s004.docx]

| Table S3. Unadjusted odds ratios and 95% confidence intervals of associations between demographic and WASH covariates and viral, bacterial, protozoal, and soil-transmitted helminth (STH) enteric infections, Saravane Province, Lao PDR, 2017 | | | | |
| --- | --- | --- | --- | --- |
|  | **Virus^1^**  (*n*=827) | **Bacteria^2^**  (*n*=833) | **Protozoa^3^**  (*n*=890) | **STH^4^**  (*n*=889) |
| Child <5 years (ref: adult) | 1.32 (0.85, 2.06) | 0.92 (0.54, 1.56) | ***3.77 (2.42, 5.87)*** | ***0.39 (0.26, 0.59)*** |
| School-aged child (ref: adult) | 0.95 (0.61, 1.49) | 0.68 (0.41, 1.14) | ***2.37 (1.58, 3.56)*** | 0.77 (0.51, 1.17) |
| Female (ref: male) | 0.74 (0.49, 1.13) | 1.22 (0.78, 1.92) | 0.40 (0.27, 1.61) | 1.39 (0.97, 1.99) |
| Socioeconomic status | 0.91 (0.79, 1.06) | 0.97 (0.84, 1.12) | 1.03 (0.93, 1.15) | ***0.81 (0.73, 0.90)*** |
| Household population size | 0.95 (0.86, 1.05) | 0.94 (0.87, 1.01) | 1.02 (0.96, 1.09) | 1.04 (0.97, 1.11) |
| Improved toilet | 1.73 (0.84, 3.57) | 0.59 (0.30, 1.18) | 0.98 (0.58, 1.63) | ***0.48 (0.27, 0.83)*** |
| Improved drinking water source | 1.31 (0.69, 2.52) | 0.64 (0.35, 1.14) | 0.84 (0.53, 1.33) | 1.25 (0.74, 2.10) |
| Basic handwashing facility | ***0.48 (0.28, 0.84)*** | 0.89 (0.51, 1.54) | 0.88 (0.59, 1.30) | ***0.51 (0.34, 0.76)*** |
| Household animal ownership | 1.20 (0.33, 4.38) | 1.26 (0.43, 3.73) | ***3.16 (1.47, 6.80)*** | 2.04 (0.82, 5.06) |
| Improved toilet coverage^5^ | 1.02 (0.86, 1.20) | 1.06 (0.94, 1.20) | 1.00 (0.91, 1.11) | ***0.80 (0.71, 0.89)*** |
| Improved drinking water coverage^5^ | 0.98 (0.87, 1.11) | 0.97 (0.89, 1.05) | 0.95 (0.88, 1.02) | 1.08 (0.98, 1.20) |
| Basic handwashing facility coverage^5^ | 1.05 (0.87, 1.28) | 1.06 (0.92, 1.22) | 1.03 (0.91, 1.16) | 1.01 (0.86, 1.20) |
| All models include random intercepts at the village and household levels to account for clustering.  ***Bold italicized*** associations indicate statistical significance at *p*<0.05  ^1^ Virus includes one or more of the following pathogens: astrovirus, adenovirus, norovirus GI, norovirus GII, rotavirus, or sapovirus. ^2^ Bacteria includes one or more of the following pathogens: *Aeromonas*, *C. difficile*, *C. jejuni*, EAEC, EHEC, EPEC (typical or atypical), LT- or ST-ETEC, *Shigella* spp./EIEC, or *Salmonella.* ^3^ Protozoa includes one or more of the following pathogens: non-hominus and non-parvum *Cryptosporidium* spp., *C.* *hominus*, *C.* *parvum*, *E. histolytica*, and *G. intestinalis.* ^4^ Soil-transmitted helminths (STH) includes one or more of the following helminths: hookworm (*N. americanus* and/or *A. duodenale*)*, A. lumbricoides*, *T. trichiura*, or *S. stercoralis.*  ^5^WASH covariate coverage is interpreted as the change in odds of infection per 10% increase in WASH covariate coverage at the village level | | | | |
